# Reliable Estimation of CD8 T Cell Inhibition of *In Vitro* HIV-1 Replication

**DOI:** 10.3389/fimmu.2021.666991

**Published:** 2021-06-30

**Authors:** Yinyan Xu, Ann Marie Weideman, Maria Abad-Fernandez, Katie R. Mollan, Sallay Kallon, Shahryar Samir, Joanna A. Warren, Genevieve Clutton, Nadia R. Roan, Adaora A. Adimora, Nancie Archin, JoAnn Kuruc, Cynthia Gay, Michael G. Hudgens, Nilu Goonetilleke

**Affiliations:** ^1^ Department of Microbiology & Immunology, University of North Carolina at Chapel Hill School of Medicine, Chapel Hill, NC, United States; ^2^ Department of Biostatistics, University of North Carolina at Chapel Hill, Chapel Hill, NC, United States; ^3^ Center for AIDS Research, School of Medicine, University of North Carolina at Chapel Hill, Chapel Hill, NC, United States; ^4^ Department of Epidemiology, Gillings School of Global Public Health, University of North Carolina, Chapel Hill, NC, United States; ^5^ School of Medicine and UNC HIV Cure Center, University of North Carolina at Chapel Hill School of Medicine, Chapel Hill, NC, United States; ^6^ Department of Urology, University of California San Francisco, San Francisco, CA, United States; ^7^ Gladstone Institute of Virology and Immunology, San Francisco, CA, United States

**Keywords:** HIV, VIA, p24, JRCSF, ROC, CD8, CD4, T-cell

## Abstract

The HIV-1 viral inhibition assay (VIA) measures CD8 T cell-mediated inhibition of HIV replication in CD4 T cells and is increasingly used for clinical testing of HIV vaccines and immunotherapies. The VIA has multiple sources of variability arising from *in vitro* HIV infection and co-culture of two T cell populations. Here, we describe multiple modifications to a 7-day VIA protocol, the most impactful being the introduction of independent replicate cultures for both HIV infected-CD4 (HIV-CD4) and HIV-CD4:CD8 T cell cultures. Virus inhibition was quantified using a ratio of weighted averages of p24+ cells in replicate cultures and the corresponding 95% confidence interval. An Excel template is provided to facilitate calculations. Virus inhibition was higher in people living with HIV suppressed on antiretroviral therapy (n=14, mean: 40.0%, median: 43.8%, range: 8.2 to 73.3%; p < 0.0001, two-tailed, exact Mann-Whitney test) compared to HIV-seronegative donors (n = 21, mean: -13.7%, median: -14.4%, range: -49.9 to 20.9%) and was stable over time (n = 6, mean %COV 9.4%, range 0.9 to 17.3%). Cross-sectional data were used to define 8% inhibition as the threshold to confidently detect specific CD8 T cell activity and determine the minimum number of culture replicates and p24+ cells needed to have 90% statistical power to detect this threshold. Last, we note that, in HIV seronegative donors, the addition of CD8 T cells to HIV infected CD4 T cells consistently increased HIV replication, though the level of increase varied markedly between donors. This co-culture effect may contribute to the weak correlations observed between CD8 T cell VIA and other measures of HIV-specific CD8 T cell function.

## Introduction

CD8 T cells detect and clear virus-infected cells using both cytolytic and non-cytolytic mechanisms. There is significant evidence both in humans and animal models, that CD8 T cells contribute to the control of human immunodeficiency virus (HIV). Within weeks of HIV infection, high frequencies of HIV-specific CD8 T cells are detected in the blood. The immune pressure exerted by HIV-specific CD8 T cells is reflected in the rapid emergence of virus escape mutations, particularly from immunodominant HIV-specific T cell responses (1). Also, certain HLA alleles are overrepresented in individuals who naturally control HIV infection. However, standard measurements of specific CD8 T cell functions correlate poorly with HIV viremia, possibly because assays measure only one or handful of CD8 T cell functions. A CD8 T cell virus inhibition assay (VIA) measures the *in vitro* ability of CD8 T cells to inhibit HIV-1 replication in autologous CD4 T cells. These assays capture the full range of CD8 T cell anti-viral activity as well as better represent HIV-derived peptides presented by infected CD4 T cells. VIA are increasingly used in clinical studies but mostly as an exploratory assay because the complex co-culture of the VIA has substantial assay variability and a limited dynamic range.

Different VIAs have been reported (2–12). VIAs have differed in length of CD8:CD4 T cell culture periods (6-13 days), purity of CD4 cultures (isolated CD4 T cells or CD8 depleted PBMCs), HIV strains (laboratory strains, isolates, reporter viruses) and read-outs of virus inhibition (p24 ELISA, intracellular measurement of p24, luciferase reporter expression and viral *gag* RNA). While there has been limited cross-laboratory validation, across reported VIAs, researchers have consistently observed that CD8 T cells from people living with HIV (PLWH) mediate greater and broader HIV inhibition than cells from seronegative individuals. Hancock and colleagues, using an intracellular p24 read-out, observed correlations between %viral inhibition and subsequent CD4 decline suggesting the utility for this assay for clinical testing of T cell-inducing vaccines and therapeutics (9). Here, we apply statistical approaches to compute percent p24 (%p24) inhibition in a modified VIA to assess assay reproducibility. These analysis approaches used weighted averages and calculated confidence intervals to quantify CD8 T cells virus inhibition across independent cultures. We observed improved reproducibility and a larger assay dynamic range. More broadly, the statistical methods applied are useful for the analysis of low frequency populations routinely studied in immunology. Virus inhibition was measured in a longitudinal cohort to examine how CD8 T cell-mediated virus inhibition varied over time in PLWH on antiviral therapy (ART). We found that in PLWH on ART, CD8 T cell virus inhibition was largely stable over time, supporting the use of this assay and, or analysis methods to examine therapeutic interventions.

## Materials and Methods

### Study Samples and Ethics Statement

Peripheral blood mononuclear cells (PBMCs) were isolated from PLWH receiving ART (HIVART) and seronegative individuals [healthy donor (HD)]. All HIVART participants were receiving stable standard-of-care ART and had maintained plasma HIV-1 RNA < 50 copies/ml and a CD4 T cell count of > 300/μl for at least 6 months before enrollment. All experimental protocols were approved by local Institutional Biomedical Review Boards (ethics numbers: 14–0741, 11–0228, 13–3613, 12-1660, and 10-01330) and performed in accordance with the relevant guidelines. HD used for assay standardization were recruited by the UNC CFAR HIV/STD Laboratory Core (IRB 96-0859, http://unccfar.org/portfolio/hiv-std-laboratory-core/) and New York Blood Center (https://nybloodcenter.org).

### Virus

The HIV-1 JRCSF infectious clone (pYK-JRCSF, #2708) was used in all presented data All viruses were propagated in HEK293T cells following plasmid transfection using Lipofectamine Plus reagent (Invitrogen Life Sciences, US) according to the manufacturer’s recommendations. Virus was collected from cell culture supernatant 48 hr post-transfection. JRCSF virus titers were determined by the TCID50. A luciferase assay was performed using TZM-bl cells with the Bright-Glo luciferase assay kit (Promega, USA), and the TCID50 value was determined by using the Reed-Muench method (13, 14). JRCSF titers ranged from 6.2 to 6.5 log10 TCID50/ml.

### Isolation, Culture and HIV-1 Infection of CD4 T Cells

Cryopreserved PBMCs were thawed (Day 0) using Benzonase (25IU/ml of culture media) and rested overnight (18-20 hr) at 37°C. The next day (Day 1) PBMCs were counted using a Muse^®^ Cell Counter (Millipore Sigma, US) in accordance with manufacturer’s instructions. Samples were counted independently, 2×, then results were averaged. CD4 T cells were isolated from the PBMC by negative selection (MACS, Milteny-Biotec 130-096-533). Cell viability was typically > 95% and purity was > 96% after the isolation. CD4 T cells were resuspended at 1 to 2 ×10^6^/ml in R-10^+^ media (RPMI-1640 + 10%FBS + L-Glu + Pen/Strep + HEPES + Sodium pyruvate) in a 6-well plate (5× 10^6^/well maximum) for 72 hr +/- 3 hr, stimulated with 3 to 5 μg/ml of phytohaemagglutinin (PHA, L8902-5MG, Sigma-Aldrich, USA), IL-2 (20 IU/ml) and IL-7 (5ng/ml) (further details in **Supplementary Material 1**). Note, 3μg/ml PHA was used to stimulate CD4 T cells from HD and 5 μg/ml for stimulation of cells from HIVART participants. Generally, the cell recovery after 72 hr (Day 4) stimulation ranged between 70-120% of the input cells, and viability ranged between 70-90%.

After 72 hr, cultured CD4 T cells were washed in 10ml R10^+^ media 3× and counted. A total of 0.1 ×10^6^ cells were set aside to be used as uninfected control cells. Cells were infected with HIV-1 JRCSF at 5x 10^6^/ml in a 15ml Falcon tube (maximum number of cells and volume used for infection/15ml Falcon tube was 2× 10^6^ and 0.4ml, respectively) by spinoculation for 2hr at 27°C in 1200*g* with IL-2 (20 units/ml) and Polybrene (4μg/ml) (NC9840454, Santa Cruz Biotechnology), at MOI 0.03.

### Isolation of CD8 T Cells

PBMCs were thawed as described above on Day 3. The next day (Day 4), PBMCs were counted independently, 2×, then results averaged. CD8 T cells were isolated from the PBMCs by positive selection in accordance with manufacturer’s instructions (MACS, Milteny-Biotec, 130-045-201). Cell viability was typically > 90% with purity > 98%.

### CD4:CD8 T Cell Co-Culture

Following spin-oculation, CD4 T cells were washed 3× in R10+. JRCSF-superinfected CD4 T cells and isolated CD8 T cells were counted independently, 2×, then results averaged. CD4 T cells were resuspended at 10^6^ cells/ml and CD8 T cells at 2×10^6^ cells/ml. To minimize cell loss from transferring cells, cell cultures were set-up in 5ml round bottom FACS tubes (12x75 mm style, Falcon, 352058) and subsequent intracellular staining at Day 7 performed in the same FACS tubes. For each participant examined, the following cell cultures, typically 13 in total, were established. All cells were cultured in R-10+ supplemented with 20 units/ml IL-2.

#### HIV-CD4:CD8 T Cell Co-Culture

Five to 6 co-cultures of CD8 and HIV-infected CD4 T cells at a ratio of 2:1 were set up. To minimize the variation, CD8 and CD4 T cells were first combined 2:1 and then divided across FACS tubes in a total volume of 200 µl/tube containing 0.3×10^6^ cells.

#### HIV-CD4 T Cell Culture

Five to six cultures of HIV-infected CD4 T cells alone were cultured in a total volume of 200 µl/tube containing 0.1×10^6^ cells.

#### CD4 T Cell Only

A single culture of uninfected CD4 T cells in a total volume of 200 µl/tube containing 0.1×10^6^ cells was established to detect endogenous virus replication.

### p24 Intracellular Staining

We adapted the cost-effective p24 intracellular staining protocol of Yang and colleagues (5). Highly statistically significant correlations have been reported between p24 intracellular staining and p24 culture supernatant levels as measured by ELISA (2, 5) and *gag* RNA measured by quantitative PCR (15); measures used in other viral inhibition assays. In this assay, cells were harvested at Day 7 and stained first with Zombie NIR™ Fixable Viability Kit (Biolegend, 423106), fixed with 4% paraformaldehyde (PFA)/lysolecithin (20 μg/ml) at RT, then resuspended in cold 50% methanol for 15min. Further permeabilization was achieved with 0.1% Nonidet P-40, and cells were then stained with antibodies to p24 antigen (KC-57- RD1, Beckman Coulter, 6604667) followed by antibodies to CD3, CD4 and CD8 receptors (conjugated to BV421, BD Biosciences; AF421, Biolegend and BV510, Biolegend respectively). Samples were acquired on a Fortessa and analyzed using FlowJo (version X10.0.7r2). Compensation controls were prepared for each fluorochrome using anti‐mouse Ig, κ compensation particles (552843, BD™ CompBead). (Gating strategies illustrated in **Supplementary 1**). The frequency of infected CD4 T cells was defined as the percentage of HIV-1 p24+ cells among live, single CD3+ CD8^negative^ lymphocytes using the uninfected CD4 cell culture that had a p24+ frequency of < 0.1 across all assays, to set the p24 gate.

### IFN-γ ELISpot

Two sets of HIV peptides were generated (Sigma-Genosys, USA): 18-mer peptides overlapping by 10 amino acids were synthesized (Sigma-Genosys, USA) to match the HIV Clade B consensus sequence (386 peptides) and previously defined HIV CD8+ optimal peptides (9- to 11-mer peptides) (16). Optimal CD8 T cell peptides describe previously defined HIV epitopes were grouped by protein, 109 Gag/Nef (CTL-A) peptides or 103 non-Gag/Nef (CTL-B) peptides. A reactive/positive T cell response to a peptide pool was defined as the average of replicate wells > 30 SFU/10^6^ PBMC and 4× the average of mock wells (16). Zero values were not accepted in any replicate of antigen-stimulated wells. No data were excluded due to high background.

### Polyfunctional Intracellular Staining

Intracellular cytokine staining (ICS) was performed as previously described (17). Similar to the VIA assay, cells were cultured and stained in 5ml round bottom FACS tubes. Briefly, a pool of peptides was added to 1 × 10^6^ PBMCs in 0.4ml at a final peptide concentration of 1.0 μg/ml. Cells were immediately stained with CD107a-APC, monensin/Brefeldin A and cultured for 6 hr at 37°C, 5% CO_2_. Next, cells were stained with Zombie NIR viability dye at room temperature for 20 min, then labeled with CD3, CD4, CD8 (conjugated to PerCP-Cy5.5, Biolegend; PE-Cy5, Biolegend and BV510, Biolegend respectively); and BV650-conjugated CD14, 16, 19 and 56 (dump channel, Biolegend) at room temperature for 15 min. Cells were fixed, permeabilized and stained intracellularly with IFN-γ, TNFα, MIP-1β, perforin (conjugated to PE, Biolegend; PE-Dazzle 594, Biolegend; PE-Cy7, BD Biosciences; BV421, Biolegend respectively). In all assays, fluorescence minus one controls (FMO) were used for gating of CD107a and cytokines. Samples were acquired on a Fortessa and analyzed using FlowJo (version X10.0.7r2). Compensation controls were prepared for each fluorochrome using anti‐mouse Ig, κ compensation particles (552843, BDTM CompBead). For all cultures, >30,000 CD8 positive events were acquired and in the final gate, a minimum of 15 events.

## Statistical Methods

### Estimating CD8 T Cell-Mediated Virus Inhibition Across Replicates

Percent CD8 T cell-mediated virus inhibition is estimated by


(1) $$\text{\% }Inhibition=\;\left(\frac{f_{HIV-CD4\;}\;-\;f_{HIV-CD4+CD8}}{f_{HIV-CD4\;}}\right)\times 100\%=\left(1-\frac{f_{HIV-CD4+CD8}}{f_{HIV-CD4\;}}\right)\times 100\%$$

where the ratio of *f_HIV_*_–_ *_CD_*_4+_ *_CD_*_8_ to *f_HIV_*_–_ *_CD_*_4_ is interpreted as an estimated relative risk (RR). This relative risk is a ratio of two composite frequencies; the numerator (*f_HIV_*_–_ *_CD_*_4+_ *_CD_*_8_) is the composite frequency of p24+ cells in superinfected CD4 T cells co-cultured with CD8 T cells, and the denominator (*f_HIV_*_–_ *_CD_*_4_) is the composite frequency of p24+ cells in CD4 T cells cultured alone.

These composite frequencies were computed by taking a weighted average of p24+ frequencies in CD4 T cells across all replicates using


(2) $$f=\frac{\sum_{i=1}^{n}w_{i}p_{i}}{\sum_{i=1}^{n}w_{i}}=\frac{w_{1}p_{1}+w_{2}p_{2}+\ldots +w_{n}p_{n}}{w_{1}+w_{2}+\ldots +w_{n}},$$

where the weights, *w_i_*, are the CD4 T cells in replicates 1 through n, and the frequencies (i.e., proportions or percentages divided by 100), *p_i_*, are the frequency of p24+ cells in superinfected CD4 T cells in each of these replicates. Equation (2) is equivalent to taking the sum of all p24+ cells across all replicates and then dividing by the sum of all CD4 T cells across replicates. To produce two separate composite frequencies, this would be done separately for superinfected CD4 T cells cultured alone and for superinfected CD4 T cells co-cultured with CD8 T cells. See **Supplementary Material 2** for this alternate formula.

For example, assume data for 3 replicates has been collected for a single participant. In superinfected CD4 T cells cultured alone, the participant has 23,000, 25,000, and 20,000 CD4 cells. The frequency of p24+ cells for these 3 replicates is 0.040 (4.0%) or 920 of 23,000 cells, 0.044 (4.4%) or 1,100 of 25,000 cells, and 0.047 (4.7%) or 940 of 20,000 cells, respectively. In co-cultures of infected CD4 T cells with CD8 T cells, the participant has 17,000, 18,000, and 15,000 CD4 T cells. The frequency of p24+ cells for these wells is 0.030 (3.0%) or 510 of 17,000 cells, 0.025 (2.5%) or 450 of 18,000 cells, and 0.023 (2.3%) or 345 of 15,000 cells, respectively.

Then, the frequencies needed for Equation (2) are calculated as


(3) $$f_{HIV-CD4+CD8}=\frac{w_{1}p_{1}+w_{2}p_{2}+w_{3}p_{3}}{w_{1}+w_{2}+w_{3}}=\frac{\left(17,000\right)\left(0.030\right)+\left(18,000\right)\left(0.025\right)+\left(15,000\right)\left(0.023\right)}{\left(17,000\;+\;18,000+15,000\right)}\approx 0.0261\;$$

and as


(4) $$f_{HIV-CD4}=\frac{w_{1}p_{1}+w_{2}p_{2}+w_{3}p_{3}}{w_{1}+w_{2}+w_{3}}=\frac{\left(23,000\right)\left(0.040\right)+\left(25,000\right)\left(0.044\right)+\left(20,000\right)\left(0.047\right)}{\left(23,000\;+\;25,000+20,000\right)}\approx 0.0435.$$

Percent inhibition can then be calculated by substituting the two frequencies calculated in Equations (3) and (4) into Equation (1) to obtain


(5) $$\text{\% }Inhibition\approx \left(1-\frac{0.0261\;}{0.0435}\right)\times 100\%=40.0\%.$$

A simulation study was used to investigate several other weighting approaches including weights of *w_i_* = 1 (simple arithmetic mean) and weights of $w_{i}=1/{\hat{\sigma }}_{i}^{2}$ (inverse variance weighting), where ${\hat{\sigma }}_{i}^{2}$ represents the variance estimate of the estimated p24+ frequency for the *i^th^* replicate. The p24+ cells were assumed to follow a binomial distribution, *Binomial*(*N_i_*, *p_i_*), where *N_i_* represents the number of CD4 T cells and *p_i_* represents the frequency of p24+ cells for the *i^th^* replicate. Thus, *p_i_* was estimated by maximum likelihood as the ratio of p24+ cells to CD4 T cells with variance estimated by ${\hat{\sigma }}_{i}^{2}=\frac{{\hat{p}}_{i}\left(1-{\hat{p}}_{i}\right)}{N_{i}}$ , where ${\hat{p}}_{i}$ represents the maximum likelihood estimate of *p_i_*. Although inverse variance weighting is optimal in a statistical sense (18), simulations involving the experimental data resulted in approximately equal performance (measured by mean squared error) when weighting with CD4 T cells [Equation (2)] *versus* weighting with the inverse variance of the p24+ frequency.

### 95% Confidence Interval for Percent Virus Inhibition

Uncertainty of the estimates of percent inhibition can be quantified using the following 95% confidence interval (CI).


(6) 95% CI=(Lower Limit, Upper Limit)

with lower and upper limits calculated using


(7) $$Lower\;Limit=100\%\left\{1-\exp\left[\ln\left(\frac{f_{HIV-CD4+CD8}}{f_{HIV-CD4}}\right)+1.96\times SE\right]\right\}$$

(8) $$Upper\;Limit=\;100\%\left\{1-\exp\left[\ln\left(\frac{f_{HIV-CD4+CD8}}{f_{HIV-CD4}}\right)-1.96\times SE\right]\right\}$$

where *exp*(·) is the exponential function and *ln*(·) is the natural logarithm. The standard error of $\ln\left(\frac{f_{HIV-CD4+CD8}}{f_{HIV-CD4}}\right)$ , the natural logarithm of a relative risk, is estimated (19) as


(9) $$SE=\sqrt{\frac{1}{{\text{c}}_{1}}-\frac{1}{{\text{c}}_{2}}+\frac{1}{{\text{c}}_{3}}-\frac{1}{{\text{c}}_{4}}}$$

where the totals (*c_i_*) in Equation (9) are calculated across all replicates as

$c_{1}=\;total\;p24+\;cells\;in\;superinfected\;CD4\;T\;cells\;cultured\;alone,$

$c_{2}=\;total\;superinfected\;CD4\;T\;cells\;cultured\;alone,$

$c_{3}=\;total\;p24+\;cells\;in\;superinfected\;CD4\;T\;cells\;co‐cultured\;with\;CD8\;cells,\;and$

$c_{4}=\;total\;superinfected\;CD4\;T\;cells\;co‐cultured\;with\;CD8\;cells.$

The SE approximation assumes that the totals (*c_i_*) for Equation (9) are sufficiently large. As a worked example, to compute the 95% confidence interval for % inhibition using the data in the previous section, the cell counts are first totaled to give:

$c_{1}=\;920\;+\;1,100\;+\;940\;=\;2,960,$

$c_{2}=\;23,000\;+\;25,000\;+\;20,000\;=\;68,000,$

$c_{3}=\;510\;+\;450\;+\;345\;=\;1,305,\;and$

$c_{4}=\;17,000\;+\;18,000\;+\;15,000\;=\;50,000.$

These totals are then substituted into Equation (7) (lower limit) and Equation (8) (upper limit) to give


$$\begin{array}{l} Lower\;Limit\approx 100\%\times \{1-exp[log\left(\frac{0.0261}{0.0435}\right)\\ +1.96\times \sqrt{\frac{1}{2,960}-\frac{1}{68,000}+\frac{1}{1,305}-\frac{1}{50,000}}]\} \end{array}$$

$$\begin{array}{l} Upper\;Limit\approx 100\%\times \{1-exp[log\left(\frac{0.0261}{0.0435}\right)\\ +1.96\times \sqrt{\frac{1}{2,960}-\frac{1}{68,000}+\frac{1}{1,305}-\frac{1}{50,000}}]\}. \end{array}$$

Finally, substituting these lower and upper limits into Equation (6) and solving gives


(12) 95% CI≈(36.0, 43.7)%.

For this particular participant, we would report a percent inhibition of 40.0% (95% CI: 36.0%, 43.7%). It is important to note that these confidence intervals are not expected to be symmetric around the estimate due to the exponential function (i.e., non-linear transformation).

Per usual, the (frequentist) confidence interval for a particular sample may not contain the true value for percent inhibition. If data were collected on a large number of participants (e.g., 1,000 participants), then approximately 95% (or 950) of the confidence intervals computed using Equation (6) should contain the true value. Simulations with 1,000 iterations were used to calculate an empirical estimate of the coverage probability of the confidence interval (**Figure 2C**). These simulations demonstrate that the mean coverage probability (dashed red line) approximates the theoretical value of 95%.

### Excel Template With Formulas

An Excel template, titled “VIA Replicate Calculator” with formulas for computing percent CD8 T cell-mediated virus inhibition [Equation (1)] and associated 95% CI [Equation (6)] can be downloaded from our GitHub repository at (https://github.com/glab-hiv/via). The first worksheet in this template provides detailed instructions on how to dynamically apply these formulas to raw data. The formulas are written to appropriately update dependent on the participant ID and culture conditions, so it is not necessary that the user modify cell references separately for each participant. To use this calculator, users will paste their data into the “Main data” worksheet and then follow the instructions on the “Instructions” worksheet to obtain estimates for % inhibition and a corresponding 95% CI.

An Excel file titled **Supplementary Material 3** “Master Supplementary”, also found at (https://github.com/glab-hiv/via), includes a sample dataset that contains only the columns necessary to compute virus inhibition and associated 95% CI. Users should employ a similar format for their raw data when using the template **Supplementary Material 4** “VIA Replicate Calculator”. Detailed instructions are provided in Excel template.

### Simulating Data to Predict Assay Performance

To evaluate assay performance (for estimation of % inhibition) in future datasets, participant data was used to stochastically generate CD4 T cell and p24+ cells. To account for both clustering and overdispersion, a negative binomial mixed-effects model (with random effect for participant ID) was fit to the CD4 count data. Parameters from this model were used to stochastically generate CD4 counts from a negative binomial distribution. Lastly, the p24+ counts were stochastically generated from a binomial distribution, *Binomial*(*N_i_*, *p_i_*), where *N_i_* are the simulated CD4 T cells and *p_i_* are the prespecified p24+ frequencies for the *i^th^* replicate.

## Results

### Inter-Culture Variability Impacts the Estimates of CD8 T Cell Virus Inhibition

**Figure 1:**
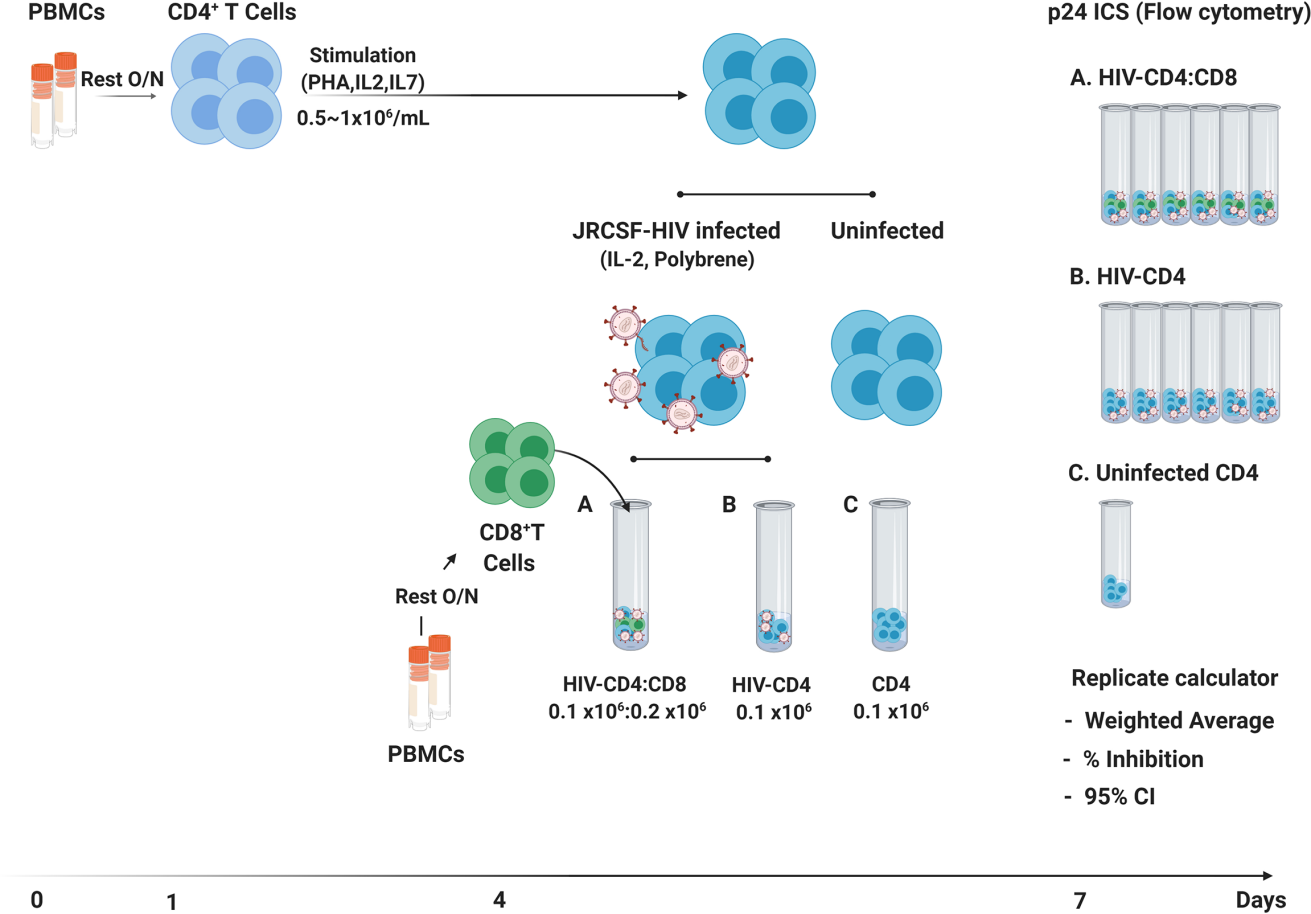
Schematic of 7-day VIA. Following an overnight rest (Day 0), CD4 T cells isolated by negative bead selection (Day 1) are activated in bulk culture for 3 days. On day 4, CD4 T cells are infected with HIV-JRCSF by spin-oculation with addition of Polybrene™ and thawed CD8 T cells are isolated by positive bead selection from autologous PBMCs. Independent cultures of CD4 T cells not JRCSF infected (n = 1), JRCSF infected CD4 T cells (n = 6) and JRCSF-CD4 T cells + CD8 T cells in 1:2 (n = 6) are set up in round-bottom flow-cytometry tubes and then cultured for another 3 days. On Day 7, all cultures (13 per participant) are stained and acquired by flow cytometry to measure intracellular p24+ frequency in CD4 (CD3^+^CD8^-^) T cells. Flow data are exported to the VIA replicate calculator (**Supplementary Material 4**) for automated calculation of weighted averages across replicate cultures, % virus inhibition and 95% confidence intervals.

A schematic of the VIA protocol used in this study is provided in **Figure 1** with a detailed protocol provided in **Supplementary Material 1**. This protocol is a modified version of previously published VIA protocols in which HIV infection is measured by intracellular staining of CD4 T cells for HIV Gag p24 (2, 5). We used CCR5-tropic HIV JRCSF in these studies to mimic both the level of cell infection and more common co-receptor usage observed following *in vitro* infection with autologous or transmitted/founder viruses (20–22).

**Figure 2:**
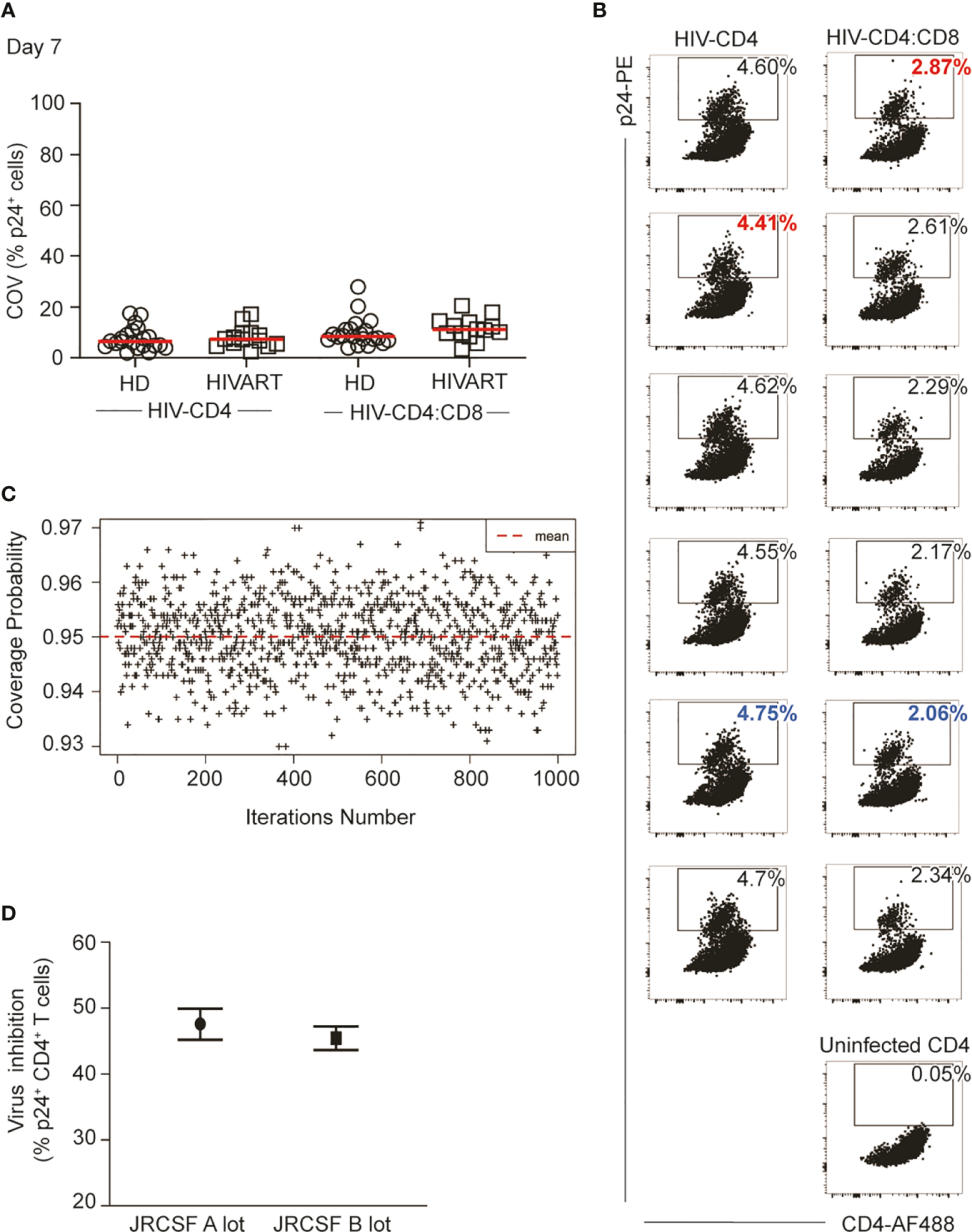
Replicate cell cultures improve assay reproducibility. **(A)** Coefficient of variation (COV) in %p24+ CD4 T cells between independent culture replicates of either HIV-CD4 or HIV-CD4:CD8 T cell co-cultures. Each symbol represents data from 1 study participant (HD in circles, HIVART in squares), and the red line indicates the group mean. **(B)** %p24+ cells measured by intracellular cytokine staining at Day 7 in HIV-CD4, HIV-CD4:CD8 and uninfected CD4 T cell cultures in patient ID 231 (data in **Supplementary Material 3**, Master Supplementary). The blue identifies the highest HIV-CD4 and the lowest HIV-CD4:CD8%p24 replicate value. Conversely, the red text identifies the lowest HIV-CD4 and the highest HIV-CD4:CD8 replicate value. **(C)** Empirical estimate for the true coverage probability of the confidence interval given in Equation (6). For fixed p24+ frequency, data were randomly sampled for 1000 participants (see Statistical Methods, subsection Simulating Data to Predict Assay Performance) using six replicates per participant. Using Equation (6), a confidence interval was computed for each estimate of percent inhibition. The proportion of intervals containing the known value was recorded, and this process was repeated 1000 times for different p24+ frequencies. **(D)** Independent measurements (3 months apart) of CD8 T cell HIV inhibition in a donor using different JRCSF stocks. HD, healthy; HIV seronegative donor; HIVART, PLWH durably suppressed with ART.

**Supplementary Material 1** details modifications made to the protocol to minimize cell loss and improve assay reproducibility. Here we focus on a relatively simple yet impactful change to the protocol, specifically the introduction of independent culture replicates for both the HIV-CD4 and HIV-CD4:CD8 T cell co-cultures. Overall, for 6 cultured replicates, the coefficient of variation (%COV) of the frequency of p24 positive cells was less than 20% for the majority of participants (n=70, mean 9.20%, range 2.04 to 27.9%) in either HIV-CD4 T cell or HIV-CD4:CD8 T cell co-cultures in both healthy donors (HD) and PLWH on ART (**Figure 2A**). However, this level of variation across cultures can considerably impact the % inhibition that results if replicate cultures are not performed. This is exampled in **Figure 2B** which shows data for Participant ID (PID) 231. The %COV across replicates for PID231 was 2.5% for HIV-CD4 and 12.5% for HIV-CD4:CD8 culture replicates. However, calculating % inhibition using the highest p24 frequency in the HIV-CD4 T cell culture and lowest p24 frequency in HIV-CD4:CD8 co-culture (red text) or vice versa (blue text), resulted in a % inhibition of 34.9% and 56.6%, respectively. Differences in % inhibition following independent repeats of single-culture assays were even greater, producing up to 40% difference in % inhibition (data not shown).

### Revised Methods to Calculate Virus Inhibition

The inclusion of replicate co-cultures to minimize intra-assay variability impacts the calculation of % inhibition in two ways. First, for each replicate, different numbers of cells were acquired (all cells were acquired to maximize cell yield). To account for the different contributions of each replicate culture to our analysis, we calculated a weighted average of p24+ cell frequencies across the HIV-CD4 T cell culture replicates. This weighted average was again calculated for the HIV-CD4:CD8 co-culture replicates. The averages were then used to calculate % inhibition as detailed in Methods.

Next, we constructed a confidence interval (CI) associated with % inhibition estimates generated from replicate cultures of both HIV-CD4 T cell cultures and HIV-CD4:CD8 T cell co-cultures. Simulations using the experimental data confirmed that 95% of the CIs contained the true value for % inhibition (**Figure 2C**). The derived 95% CI appropriately captures the variation in p24+ frequencies across both HIV-CD4 and HIV-CD4:CD8 T cell replicate cultures in our assay. In **Figure 2D**, we apply calculation of % inhibition [Equation (1)] and apply confidence intervals [from Equation (6)] to the data shown for PID 231 in **Figure 2B**. The calculated virus inhibition was 47.6% (95% CI: 45.2%, 49.9%). To examine whether these calculations improved reproducibility, we re-tested virus inhibition in PID231 three months later using a different lot of JRCSF. The calculated virus inhibition was similar at 45.5% (95% CI: 43.6%, 47.2%) indicating good reproducibility.

### CD8 T Cell Mediated HIV-1 Inhibition Is Significantly Higher in PLWH on ART Relative to Seronegative Donors

**Figure 3:**
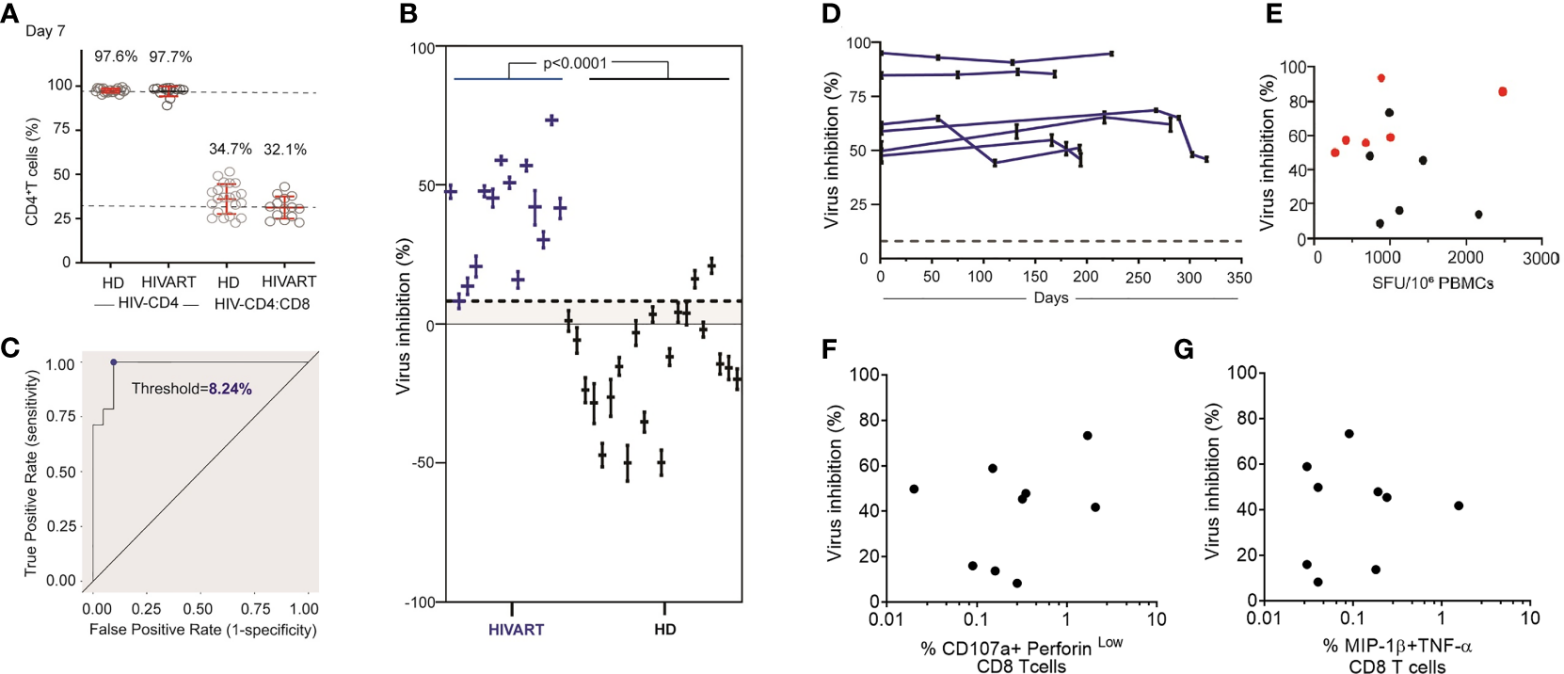
HIVART participants produce higher % inhibition of HIV than healthy donors. **(A)** %CD4 T cells at Day 7 in HIV-CD4 and HIV-CD4:CD8 T cell culture in HD and HIVART participants. Mean and SD shown in red. **(B)** Individual estimates for % inhibition [Equation (1)] and corresponding 95% CI [Equation (6)] in HD and HIVART participants. Difference in % inhibition between the groups (p < 0.0001) was tested with an two-tailed, exact Mann-Whitney test. **(C)** The threshold value (blue dot) of 8.24% CD8 T cell-mediated virus inhibition was determined by maximizing the true positive rate (probability a participant has a positive value for percent inhibition, given they are seropositive) and minimizing the false positive rate (probability a participant has positive value for percent inhibition, given that they are seronegative). This threshold value is shown as the dashed line in **(B, D)** Estimates for % inhibition [Equation (1)] and corresponding 95% CI [Equation (6)] in HIVART participants (n = 6) over time. Each line = data from 1 participant. Virus inhibition measured on Day 7 does not correlate with T cell responses **(E)** to the HIV clade B proteome measured by overnight IFN-γ ELISpot (n = 12, r = -0.03, p = 0.94; two-tailed Spearman’s rank correlation) or **(F)** to the HIV optimal epitopes (CTLA+CTLB) measuring % of CD107a+perforin^low^ CD8 T cells (n = 9, r = -0.08, p = 0.84; two-tailed Spearman’s rank correlation) by 6hr ICS or **(G)** %MIP-1β+TNF-α+ CD8 T cells (n = 9, r = -0.09, p = 0.82; two-tailed Spearman’s rank correlation); dashed line = threshold, HD = healthy, HIV seronegative donor, HIVART = PLWH durably suppressed with ART; **(E–G)** black symbol = %inhibition measured at a single timepoint as shown in **(C)**, red symbol = average % inhibition over time as shown in **(D)**.

Using this assay, CD8 T cell inhibition of JRCSF infected CD4 T cells was assessed in HD (n=21) and HIVART donors (n=14). Across the cohort, HIV infection of CD4 T cells measured at day 3 post-infection ranged from 1.08 to 15.16% (median: 5.1%). On average, 990 (range: 240 to 2,742) p24+ cells and 22,500 (range: 4,271 to 52,394) CD4 T cells were acquired per participant examined (n=35). While we did not routinely examine the purity of cell isolations, the flow-based analysis enabled analysis of the CD4:CD8 T cell ratio following co-culture. In co-culture assays, set up in a ratio of 1 CD4 target cell to 2 CD8 T cells, the Day 7 ratio remained very close to 1:2 (**Figure 3A**, %CD4 T cells in total T cells: HD T cell=34.7%, HIVART=32.1%, n=35), suggesting a high and consistent purity of CD4 and CD8 T cell isolation. These ratios also indicate that neither CD4 nor CD8 T cell populations were undergoing dynamic changes during co-culture (Days 4-7).

When % inhibition was then calculated using Equation (1), we observed that all HIVART participants produced a positive (> 0%) virus inhibition (n=14, range: 8.2% to 73.3% inhibition), consistent with our previous reports that HIVART participants maintain functional HIV-specific CD8 T cells over time (16, 23) (**Figure 3B**). The level of inhibition in HD was mostly negative (n=21, mean: -13.7%, range: -49.9 to 20.9%), though some participants produced % inhibition > 0%, suggesting false positives or non-specific CD8 T cell mediated virus inhibition (**Figure 3B**). The negative % inhibition values observed in HD reflected that higher HIV replication (i.e., higher p24+ frequencies) was observed in HIV-CD4:CD8 T cell co-cultures than HIV CD4 T cell cultures, suggesting the addition of CD8 T cells (that are not specific for HIV) enhanced HIV replication. Note, we also observed higher HIV replication in CD8-depleted PBMC compared with CD4 T cell-only cultures (**Supplementary Figure 1A**). Overall, HIVART participants produced consistently positive and higher levels of % inhibition than HD (**Figure 3B**) (n=35, p<0.0001, two-tailed, exact Mann-Whitney test).

A receiver operating characteristic (ROC) curve (**Figure 3C**) of study data demonstrates that the optimized assay can reliably predict values of specific CD8 T cell mediated virus inhibition >8% (rounded down from 8.24%). This threshold was determined by maximizing the true positive rate and minimizing the false positive rate. The estimates of within-sample sensitivity and specificity were 100.0% (14/14) and 90.5% (19/21), respectively. In other words, when this assay is repeated in the future with 6 replicates per participant, one can confidently detect HIV-specific CD8 T cell mediated inhibition for percentages greater than 8%.

### CD8 T Cell Mediated HIV-1 Inhibition Is Stable in PLWH on ART

CD8 T cell inhibition over time was measured in PLWH on ART (**Figure 3D**). Participants all initiated ART in chronic infection and at the time of VIA testing had been durably suppressed for 3.3-10.5 years. In each participant, CD4 T cell target cells from a single timepoint were cultured with CD8 T cells isolated at 4-5 timepoints over a 6-12months window. CD8 T cell inhibition was relatively stable over the time window tested, with %COV in six participants averaging 9.4%, range 0.9 to 17.3%. We examined whether % inhibition correlated with *ex vivo* IFN-γ production measured within the same timeframe (**Figure 3E**). Consistent with previous reports (10), the CD8 T cell inhibition of JRCSF did not correlate with the summed magnitude of T cell response to overlapping peptides spanning the HIV clade B proteome measured by IFN-γ ELISpot (n=12, r=-0.03, p=0.9; two-tailed Spearman’s rank correlation). Similarly, no evidence of correlation was observed between virus inhibition and either cytolytic function CD107a+perforin^low^, (n=9, r=-0.08, p=0.8; two-tailed Spearman’s rank correlation) or MIP-1β+TNF-α (n=9, r= -0.09, p=0.8; two-tailed Spearman’s rank correlation) release of HIV-specific CD8 T cells measured in 6-hour ICS (**Figures 3F, G**). Note, IFN-γ measurements were correlated between the ELISpot and ICS assay (**Supplementary Figure 1B**) (24).

### Assay Criteria Required to Provide 90% Power to Reliably Predict Virus Inhibition

A final question, which is a common challenge in flow cytometry when examining low frequency cells is, ‘What is the minimum infection rate (%p24+) and minimum cells necessary to see a “real” change in percent virus inhibition?’. Given our use of replicate cultures, we also examined how this minimum changed depending on the number of replicates, i.e., were six replicates needed if either a higher number of p24+ cells were acquired or a higher % inhibition was observed?

Assuming 20,000 CD4 T cells were acquired, simulations were constructed so that the minimum acceptable HIV infection rate was 0.5% (p24+ cells in HIV-CD4 T cells). At 90% power, for 6 replicates, a minimum infection rate of 2.43% is needed to observe a true virus inhibition of 8% (**Table 1**). Again, assuming a denominator of 20,000 CD4 T cells were acquired, this translates to a minimum of 486 p24+ cells (**Table 2**). If the number of replicates is decreased to a standard of 3 replicates per participant, a higher infection rate of 4.63% (p24+ in CD4 T cells cultured alone) is necessary (**Table 1**). This translates to a minimum of 926 p24+ cells to have 90% power to observe 8% virus inhibition (**Table 2**). Given that 20% virus inhibition is more likely to achieve a true positive threshold than 10% inhibition (reminder, % inhibition is a relative risk calculation), the absolute number of p24 cells required decreases as this threshold increases. Percent virus inhibition ≥ 30% requires that only 100 p24+ cells (our minimum assigned threshold) are acquired, whether 2 through 6 culture replicates are used.

**Table 1:** At 90% statistical power, the minimum percentage of p24+ cells (in HIV-CD4 T cell cultures) needed to observe a threshold virus inhibition of 5-30% for 2-6 replicates.

Number of Replicates	Virus Inhibition (%)						
	5	8	10	15	20	25	30
2	14.99	7.49	4.78	1.98	1.05	0.61	0.5
3	11.84	4.63	3.16	1.3	0.67	0.5	0.5
4	8.91	3.69	2.3	0.95	0.5	0.5	0.5
5	7.79	3.17	1.88	0.79	0.5	0.5	0.5
6	6.97	2.43	1.57	0.66	0.5	0.5	0.5

The threshold of 8% inhibition is the ROC-determined cut-off of specific CD8 T cell mediated virus inhibition from study data. Percentages of p24+ cells were generated using simulations (500 iterations) based on parameters from the real data. The minimum allowed percentage of HIV infection was 0.5%.

**Table 2:** At 90% power, the minimum number of p24+ cells (denominator of 20,000 CD4+ T cells) needed to observe a threshold virus inhibition of 5-30% for 2-6 replicates.

Number of Replicates	Virus Inhibition (%)						
	5	8	10	15	20	25	30
2	2999	1498	957	397	209	122	100
3	2367	926	632	259	135	100	100
4	1783	738	459	191	100	100	100
5	1559	635	375	159	100	100	100
6	1393	486	314	133	100	100	100

The minimum allowed percentage of HIV infection was 0.5%.

## Discussion

Multiple VIAs have been published, all consistently reporting higher % inhibition in HIV seropositive donors +/- ART than HIV seronegative, otherwise healthy donors (2, 4, 5, 9, 25, 26). We also observed clear differences between HIV-infected ART-suppressed donors and healthy donors in VIA measurements. Here, we identify methodological and analysis changes that could be incorporated into other protocols to improve assay reproducibility.

First, we adapted the previously described approach by Saez-Cirion et al. (3) to HIV infect isolated CD4 T cells, rather than CD8-depleted PBMC. This produced lower false-positive or non-specific virus inhibition in HD, enabling a greater dynamic range (8 to 100%) in which to detect specific % inhibition. This may be of particular benefit in therapeutic intervention studies where the goal is to detect a change, presumably an increase, in virus inhibition following vaccination or immunotherapy in PLWH. It is notable that percent inhibition was consistently negative in our HD cohort, reflecting higher HIV replication in CD4:CD8 co-cultures than CD4 T cells alone. It is unclear how the addition of non-stimulated CD8 T cells from HD facilitates increased *in vitro* HIV replication in CD4 T cells. It is possible that both increased cell density in the co-culture and cell-intrinsic factors including soluble factors may have contributed. We also observed higher HIV replication in CD8-depleted PBMCs than isolated CD4 T cell cultures, suggesting that non-CD4 T cells beyond CD8 T cells may be capable of enhancing *in vitro* HIV replication. The broad range of negative virus inhibition observed indicates inter-individual differences in how non-HIV specific CD8 T cells in HD impact HIV replication. In the absence of pre-HIV infection samples, how non-HIV specific CD8 T cells impact inhibition measurements in HIVART donors cannot be quantified. We conclude from these data that, in HIVART donors, HIV-specific CD8 T cells consistently inhibit infection *in vitro*, but the final % inhibition calculation is also impacted by non-HIV specific CD8 T cells (which occur in large excess) that simultaneously enhance virus replication.

Practical changes to the VIA, such as cell culture in flow cytometry tubes (**Supplementary Material 1** - detailed protocol), enabled increased assay throughput which is also likely to be beneficial for clinical testing. In our hands, the introduction and acquisition of independent culture replicates greatly improved assay confidence. An Excel template is provided with this manuscript to facilitate easy calculation of % virus inhibition and a corresponding 95% CI (**VIA Replicate Calculator, https://github.com/glab-hiv/via**). These calculations have a broader application in flow cytometry where replicate assays acquire different cell numbers. We recommend that investigators consider calculation of % inhibition *via* weighted averages and 95% CI using the template provided. Weighted averages and 95% CI could also be used for analysis of independent replicates in standard functional ICS.

Simple tables (**Tables 1** and **2**) are provided to help determine the level of HIV infection needed and the minimum number of p24+ cells that must be acquired to achieve 90% statistical power to detect CD8 T cell mediated inhibition of HIV replication. Again, the values produced in simulations for these tables have broader application in flow cytometry, where low frequency cells are being measured to define the minimum cell numbers and/or replicates required to have statistical power to detect a difference in functional response (e.g., frequency of cytokine producing cells).

The final protocol was used to examine CD8 T cell mediated inhibition of HIV-infected CD4 T cells in PLWH on ART over time. Consistent with our recent study examining HIV-specific T cell responses measured using *ex vivo* IFN-γ ELISpot over time (16), virus inhibition was also relatively stable (average %COV < 10%) when measured over a 6-12 month window. Also, consistent with other reports, there was no evidence of correlation between virus inhibition and the summed magnitude of T cell responses against the HIV clade B proteome or optimal HIV epitopes measured by ELISpot or cytolytic function measured by ICS. Multiple factors could contribute to this lack of correlation. This includes the different functional read-outs (3-day *in vitro* cytolytic activity in the VIA *vs ex vivo* function) and HIV sequence differences between the participant’s infecting virus, JRCSF used in the VIA and different HIV peptides sets used in *ex vivo* assays. The HIV JRCSF virus used in VIAs and consensus clade B sequence used to design peptides tested in ELISpots exhibit 94% amino acid sequence identity very likely impacting CD8 T cell recognition of epitopes. The co-culture effect, observed in HD showing higher HIV replication in CD4:CD8 co-cultures than CD4 cells, may also contribute to the weak correlations observed. Future studies will examine whether stronger correlations are observed when virus inhibition is compared to targeting of protective epitopes (27) as previously reported by Hancock et al. (10).

This work has some limitations. First, as just noted, this assay was standardized using one laboratory strain of HIV JRCSF. Other HIV viruses, including autologous participant and X4-tropic isolates, have been used for VIAs (28). These culture conditions will need to be tested against each virus by measuring % inhibition in both HD and PLWH samples to define inhibition thresholds. Our parameters for number of cells acquired and culture replicates, detailed in **Tables 1** and **2**, assumed a low virus infectivity of 0.5% cells, which can be achieved by both autologous virus and laboratory isolates. Therefore, we are confident that good reproducibility will be observed across different HIV isolates. Second, to our knowledge, this is the first study using ROC analysis to establish an 8% threshold to detect specific virus inhibition. Independent datasets are needed to fully evaluate the sensitivity and specificity of this approach. Third, independent cultures necessitate higher cell numbers. While we believe the improved reproducibility is worthwhile, this method does not preclude further iteration such as miniaturization to decrease cell numbers needed and alternate, more sensitive HIV read-outs such are measuring HIV RNA (15). We note that the flow analysis of mixed cultures that provides information on culture viability and CD4:CD8 T cell ratio is beneficial at least at the stage of assay standardization.

In summary, despite the use of purified cell populations, we observed considerable inter-culture variability in the frequency of intracellular p24+ cells in 3-day HIV-infected T cell cultures. The introduction of independent culture replicates decreased variability but required the development of new analysis methods to calculate average virus inhibition and corresponding confidence intervals. These analysis methods and associated power calculations may have broader utility for the detection of low frequency events in flow cytometry. Using these analysis methods, we show that CD8 T cell mediated HIV inhibition in PLWH on ART can be reliably measured and is largely stable over a 6-12 month period.

## Data Availability Statement

The original contributions presented in the study are included in the article/**Supplementary Material**. Further inquiries can be directed to the corresponding author.

## Ethics Statement

The studies involving human participants were reviewed and approved by University of North Carolina at Chapel Hill Biomedical Institutional Review Board (IRB) and the University of California at San Francisco IRB. The patients/participants provided their written informed consent to participate in this study.

## Author Contributions

NG conceived and designed the study. YX with assistance from MA-F, GC, SK, SS, and JW performed all experimental assays. AMW developed statistical methods with review and oversight from KM and MH. NR, AA, NA, JK, and CG provided clinical samples. YX, AMW, MA-F and NG wrote the manuscript with review from all authors. All authors contributed to the article and approved the submitted version.

## Funding

This work was supported by the National Institute of Allergy and Infectious Disease, U01 AI131310-01 (N.G) and University of North Carolina at Chapel Hill Center for AIDS Research (P30 AI050410).

## Conflict of Interest

The authors declare that the research was conducted in the absence of any commercial or financial relationships that could be construed as a potential conflict of interest.

## Supplementary Material

Supplementary Figure 1
**(A)** %p24 levels in JR-CSF infected CD4 T cells or autologous CD8-depleted PBMC at day 6 post-infection (n=6, p=0.0313; two-tailed, exact Wilcoxon signed-rank test). **(B)** Correlation between HIV-specific T cell response (CTLA+CTLB) measured in *ex vivo* IFN-γ ELISpot and %IFN-γ+ by ICS (n=8, r=0.82, p=0.0122; two-tailed Spearman’s rank correlation).
