# Corrigendum: Reliable estimation of CD8 T cell inhibition of *in vitro* HIV-1 replication

**DOI:** 10.3389/fimmu.2022.1104661

**Published:** 2023-01-03

**Authors:** Yinyan Xu, Ann Marie Weideman, Maria Abad-Fernandez, Katie R. Mollan, Sallay Kallon, Shahryar Samir, Joanna A. Warren, Genevieve Clutton, Nadia R. Roan, Adaora A. Adimora, Nancie Archin, JoAnn Kuruc, Cynthia Gay, Michael G. Hudgens, Nilu Goonetilleke

**Affiliations:** ^1^ Department of Microbiology & Immunology, University of North Carolina at Chapel Hill School of Medicine, Chapel Hill, NC, United States; ^2^ Department of Biostatistics, University of North Carolina at Chapel Hill, Chapel Hill, NC, United States; ^3^ Center for AIDS Research, School of Medicine, University of North Carolina at Chapel Hill, Chapel Hill, NC, United States; ^4^ Department of Epidemiology, Gillings School of Global Public Health, University of North Carolina, Chapel Hill, NC, United States; ^5^ School of Medicine and UNC HIV Cure Center, University of North Carolina at Chapel Hill School of Medicine, Chapel Hill, NC, United States; ^6^ Department of Urology, University of California San Francisco, San Francisco, CA, United States; ^7^ Gladstone Institute of Virology and Immunology, San Francisco, CA, United States

**Keywords:** HIV, VIA, p24, JRCSF, ROC, CD8, CD4, T-cell

In the published article, an author name was incorrectly written as Nadia Roan. The correct spelling is Nadia R. Roan.

In the published article, an author name was incorrectly written as Cindy Gay. The correct spelling is Cynthia Gay.

The authors apologize for these errors and state that they do not change the scientific conclusions of the article in any way. The original article has been updated.

## Publisher’s note

All claims expressed in this article are solely those of the authors and do not necessarily represent those of their affiliated organizations, or those of the publisher, the editors and the reviewers. Any product that may be evaluated in this article, or claim that may be made by its manufacturer, is not guaranteed or endorsed by the publisher.

