# Supplementary figures and images for: Reliable Estimation of CD8 T Cell Inhibition of In Vitro HIV-1 Replication
Source: Front Immunol. 2021 Jun 30;12:666991. doi: 10.3389/fimmu.2021.666991 (PMC8278574; doi:10.3389/fimmu.2021.666991)

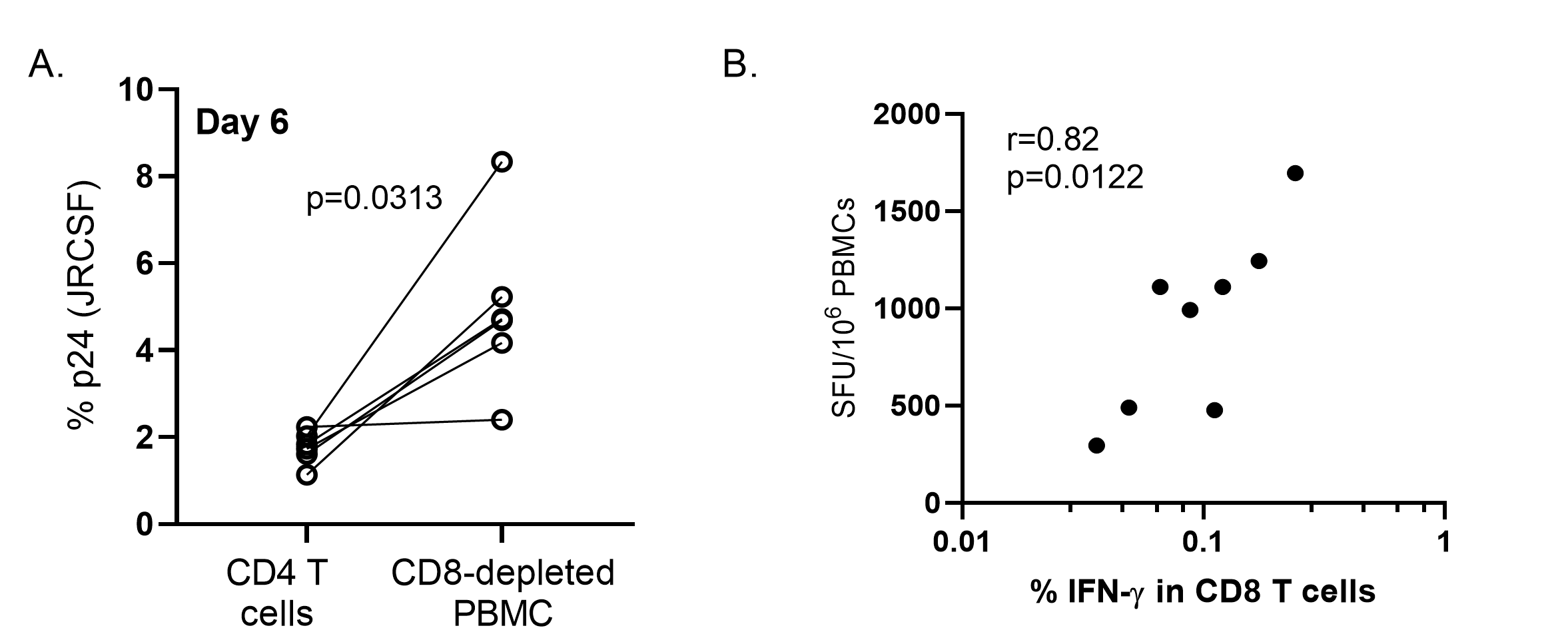

Supplement: Supplementary Figure 1 — (A) %p24 levels in JR-CSF infected CD4 T cells or autologous CD8-depleted PBMC at day 6 post-infection (n=6, p=0.0313; two-tailed, exact Wilcoxon signed-rank test). (B) Correlation between HIV-specific T cell response (CTLA+CTLB) measured in ex vivo IFN-γ ELISpot and %IFN-γ+ by ICS (n=8, r=0.82, p=0.0122; two-tailed Spearman’s rank correlation). [file Image_1.tif]
