# Supplementary material for: Reliable Estimation of CD8 T Cell Inhibition of In Vitro HIV-1 Replication
Source: Front Immunol. 2021 Jun 30;12:666991. doi: 10.3389/fimmu.2021.666991 (PMC8278574; doi:10.3389/fimmu.2021.666991)
Supplement: Supplementary file 2 [file DataSheet_1.docx]

### CD8 T cell Virus Inhibition Assay Protocol Version 1.0

Author: **Yinyan Xu**

Reference: Xu, Weideman et al Frontiers in Immunology 2021

Laboratory: Goonetilleke

Institution: University of North Carolina

Acknowledgement: With the exception of flow antibodies used, the p24 intracellular staining steps described here are as per Yang et al JIM 2013 PMID:23500782.

| **Reagents** | **Supplier** |
| --- | --- |
| Phytohaemagglutinin (PHA) | L8902-5MG, Sigma-Aldrich, USA |
| Benzonase® Nuclease | E1014, Sigma-Aldrich, USA |
| CD4 +T Cell Isolation Kit | MACS, Milteny-Biotec 130-096-533 |
| *HIV-1_JRCSF_ | HIV-1 JR-CSF Infectious Molecular Clone (pYK-JRCSF) NIH reagent program |
| IL-2 | Prometheus Proleukin, Aldesleukin, Lot # 407682M |
| IL-7 | PeproTech, 200-07-10UG |
| Polybrene | NC9840454, Santa Cruz Biotechnology |
| RPMI | [10-040-CV](https://ecatalog.corning.com/life-sciences/b2c/US/en/Media%2C-Sera%2C-and-Reagents/Classical-Media/RPMI-1640/Corning%C2%AE-RPMI-1640/p/10-040-CV), Corning® |
| FBS | 97068-091, VWR Life Science |
| L-Glutamine | Corning™ 25005CI |
| Sodium Pyruvate | Corning™ 25-000-CIR |
| Penicillin-  streptomycin | Gibco™ 15070063 |
| HEPES | Corning™ [25-060-CI](https://ecatalog.corning.com/life-sciences/b2c/US/en/Media%2C-Sera%2C-and-Reagents/Cell-Culture-Supplements/Buffers-Culture/Corning%C2%AE-HEPES/p/25-060-CI) |
| PBS | Corning™ 21-030-CM |
| BSA | Sigma A8412 |
| EDTA | Corning™ 46-034-CI |
| lysolecithin | Sigma, L4129-25MG |
| Paraformaldehyde (PFA) | Santa Cruz Biotechnology, sc-281692 |
| Methanol | Sigma, 34860-1L-R |
| Nonidet P-40 | Biotang Inc, BTBB914 |

*Store in single use aliquots at -80°C

| **Antibodies** | **Clone** |
| --- | --- |
| Zombie NIR | Fixable Viability Kit, Biolegend, 423106 |
| p24-FITC | KC57-FITC, 6604665, Beckman |
| CD3-BV421 | BD Biosciences, BDB562426 |
| CD4-AF488 | Biolegend, 317434 |
| CD8-BV510 | Biolegend, 344732 |
| anti‐mouse Ig, κ compensation beads | BDTM 552843 |
|  |  |
| **Media, Buffers (Storage)** | **Recipe** |
| R-10+  4^0^C (limit light exposure) | RPMI-1640/ 10% FBS/ 2nM L-glutamine/ 1mM sodium pyruvate/ 1x penicillin-streptomycin/ 10mM HEPES |
| R-20+  4^0^C (limit light exposure) | RPMC-1640/ 20% FBS/ 2nM L-glutamine/ 1mM sodium pyruvate/ 1x penicillin-streptomycin/ 10mM HEPES |
| Cell isolation Buffer 4^0^C | PBS/0.5%BSA/2mM EDTA |
| 20 μg/ml lysolecithin in 4% PFA #  2-8^0^C | Dissolve 10 mg lysolecithin in 500 ml of the 4% PFA solution |
| 50% Methanol/PBS  -20^0^C # |  |
| 0.1% NP-40/PBS*  2-80^0^C # | 0.1% NP-40 (Nonidet P-40, Biotang Inc, BTBB914): Add 0.5 ml NP-40 to 499.5 ml of PBS. |

**^#^***Note: The above reagent solutions have a minimum shelf life of 6 months, if stored as indicated*

| **Key Equipment** | **Comment** |
| --- | --- |
| 2 Benchtop Centrifuges | 1 temperature controlled |
| Cell counting | Recommend automated counter |
| Incubator | C02, 5% |
| Miltenyi Biotech MACS magnet |  |
| Vortex |  |
| Flow Cytometer |  |

**METHOD:**


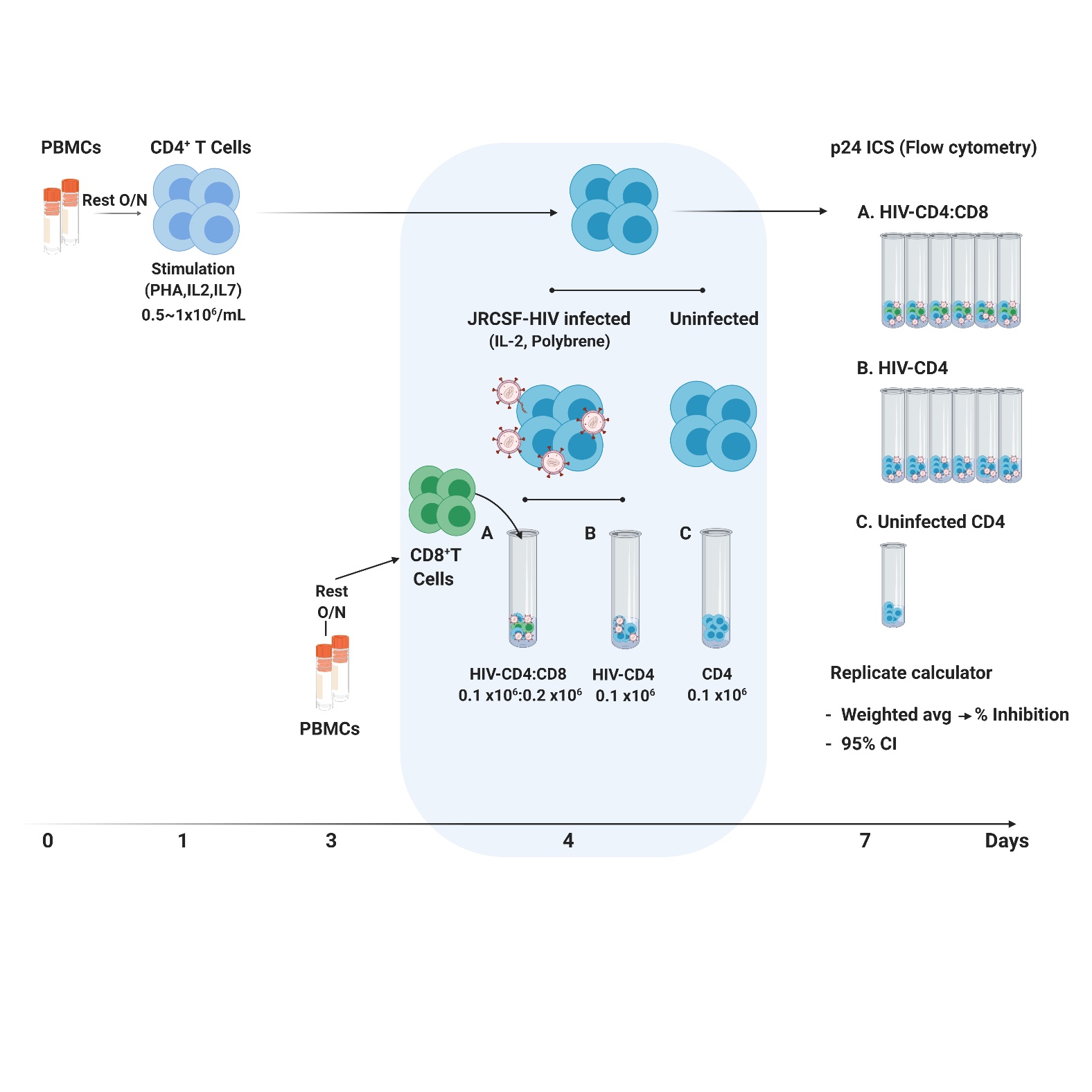


**Day 0**: **Thaw PBMCs for CD4+ T cell targets**:

1. Make 25 units/ml Benzonase in R-10+ and warm in a 37°C incubator.
2. Rapidly thaw PBMC in a 37°C waterbath, then transfer cell contents into 10ml prewarmed R10+ with benzonase (25 unit/ml), wash 3x with R-10+ and count. Resuspend at 2x10^6^ cells/ml in R20+ and place in a 37^o^C, humidified CO_2_ incubator overnight. See **Table 1** for PBMC requirements.

**Table 1**: PBMC estimates for VIA^a^

| VIA PBMC requirements ^b^ | 1 timepoint | 4 timepoints^c^ |
| --- | --- | --- |
| Targets | 10^7^ | 3-4 x10^7^ |
| Effectors (2:1) | 10^7^ | 10^7^/ timepoint |

^a^ Estimates based on JRCSF infection ranging 1-10% and PBMC viability >85%, Using 0.1x10^6^ CD4+ T cells per replicate, total 4-6 replicates.

^b^ Assume 6 CD4-HIV and 6 CD4-HIV:CD8 co-cultures and 1 uninfected control

^c^ One set of CD4-HIV-only and 1 uninfected CD4 control will be used for %inhibition calculations across multiple timepoints

*Note I: Benzonase decreases cell clumping, improving cell recovery.*

*Note II: Negative Isolation of CD4+ T cells produced lower non-specific virus inhibition in HIV-negative donors than assays performed with CD8-depleted PBMC.*

**DAY 1**: **Isolate CD4+ T cell targets**:

1. Negatively isolate CD4+ T cells from thawed PBMCs using MACS beads as per manufacturer’s instructions. A typical isolation provides >96% CD4+ T cell purity.
2. Count and suspend cells at 2×10^6^ /ml in R-10+ media containing IL-2 (20 IU/ml), IL-7 (5ng/ml) and either 5μg/ml PHA (people living with HIV+/- ART) or 3μg/ml (HIV seronegative donor).
3. Plate the cells as listed and culture for 72hr +/- 3hr at in a 37°C incubator 5% CO_2_:
   - - 5 x 10^6^ /well in 6-well plate
     - 3 x 10^6^ /well in 12-well plate
     - <1.5 x 10^6^ in 24 - or 48 -well plates

*Note I: VIA assays using CD8-depleted PBMC are reported and in our hands result in higher infectivity (%p24+ cells). However, CD4+ T cells are recommended over CD8-depleted PBMC because of improved assay reproducibility possibly due to the exclusion of CD56+ NK cells and more consistent CD4:CD8 T cell ratios.*

*Note II: IL-2+IL-7 results in higher %p24+ infection possibly due to IL-7-mediated increase of CCR5 expression on CD4+ T cells (Llano et al 2001 PMID: 11581400).*

*Note III: Final CD4+ T cell concentrations can be 1-2x10^6^ depending on volume capacity in each well. Generally, the cell recovery after 72 hrs (Day 4) stimulation ranges between 70-120% of the input cells, and viability ranges between 70-90%.*

*Note IV: The different PHA concentrations are used because PHA > 3ug/ml in seronegative donors results in significant cell loss. Cell loss following > 3ug/ml PHA stimulation is also observed in HIV+ donors but the 5ug/ml stimulation improves HIV %infection.*

**DAY 3**: **Thaw PBMCs for CD8+ T cell effectors**:

1. Make 25 unit/ml Benzonase in R-10+ and warm in a 37°C incubator.
2. Rapidly thaw PBMC in a 37°C waterbath, then transfer cell contents to in prewarmed R-10+ with benzonase (25 unit/ml), wash 3x with R-10+ and count. Resuspend in 2x10^6^/ml in R-20+ and place in a 37^o^C, humidified CO_2_ incubator overnight. See **Table 1** for PBMC needs.

**DAY 4**: **HIV infection of target CD4+ T cells**:

1. 72hr +/-3hr stimulation with PHA, harvest mitogen activated CD4+ targets from wells using a sterile disposable pipette and wash 3x with >10ml R10+ to remove PHA in culture.
2. Count cells and resuspend to 10^7^/ml in a 15ml Falcon tube in R-10+ supplemented with IL-2 at 20IU/ml.

*Note: Generally, the cell recovery after 72 hr (Day 4) stimulation ranged between 70-120% of the input cells, and viability ranged between 70-90%.*

3. Transfer 10^5^ cells to a flow cytometry tube for use as uninfected controls.

4. Infect the remaining CD4+ T cells with HIV-1_JRCSF_ (final MOI at 0.03).

- 1. Supplement R-10+ with IL-2 at 20IU/ml and Polybrene 8ug/ml
  2. Dilute virus stock in Polybrene/IL-2/R-10+ media to a 2x final concentration.
  3. Next add an equal volume of 2x virus to CD4+ targets resulting in a final cell concentration of 5x10^6^/ml. Do not exceed more than 2x10^6^ cells per 15ml Falcon tube (0.4ml).
  4. Spin-oculate at 2000rpm, 27^0^C for 2 hours with brake.
  5. Following spin-oculation, wash 3x with R-10+ to remove residual virus and polybrene.

1. Count and re-suspend cells at 1x10^6^ per ml by adding R-10+ supplemented with IL-2 at 20 IU/ml.

*Note I: Counting accuracy is critical in this step for ensuring the correct ratio of CD4+ targets to CD8+ effectors. Counting in triplicates is recommended with a %CV <10%.*

*Note II: Addition of polybrene increases JRCSF % infection by up to 3-fold but does not improve NL4.3 infection.*

**DAY 4: Prepare CD8+ T cells effectors:**

1. While CD4+ T cells are spin-oculating, isolate CD8+ T cells from rested overnight PBMC using CD8+ MACs microbeads (positive selection) following the manufacturer’s instructions.
2. Count and re-suspend isolated CD8+ T cells at 2x10^6^/ml in R-10+ with IL-2 at 20 IU/ml.

*Note: Again, counting accuracy is critical in this step for ensuring correct ratio of CD4+ targets to CD8+ effectors. Counting in triplicates and a %CV <10% is recommended.*

**DAY 4: Effector: Target co-cultures:**

1. Cell numbers permitting, set up the following labeled flow cytometry tubes:
   - 6 tubes containing 10^5^ CD4+ targets (100μl) + 100μl R10
   - 6 tube containing 10^5^ CD4+ targets (100μl) + 2x10^5^ (100μl) CD8+ T cell effectors
   - 1 tube containing 10^5^ uninfected CD4+ cells (100μl) + 100μl R10

*Note I: To minimize the variation CD8+ and CD4+ T cells were first combined 2:1 and then divided across flow cytometry tubes in a total volume of 200 µl /tube containing 3×10^5^ cells.*

*Note II: This is day 0 of HIV-1 JRCSF super-infection and effector target co-culture. Peak of infection (p24%) is at day 3.*

*Note III: If using different E:T ratios, adjust according. We recommend ensuring the final culture volume across all tubes is kept consistent.*

*Note IV: Sterile flow cytometry tubes are recommended over 48- and 96-well plates for cultures containing 1- 3x10^5^ cells because of better infectivity, less cell loss and time savings.*

# DAY 7: Intracellular HIV-specific p24 staining protocol

*Note I: Peak %p24+ cells are observed 3 days post-infection (data not shown)*

*Note I: Cell cultures are directly stained in the same flow cytometry tubes. We recommend using printed labels for flow tubes (not handwritten).*

| **Tube** | **Detail** | **Replicates** |
| --- | --- | --- |
| Live Dead control | Heat kill cells in 70°C water bath, 10min | 1 |
| Controls | CD4 FMO recommended as infection ↓ CD4 expression | 1 |
|  | Non-stained control | 1 |
| Test Samples | Un-infected control CD4-only^a^ | 1 |
|  | Infected CD4+ targets | 6^b^ |
|  | Co-culture: Infected CD4+ targets + Effector CD8+ | 6^b^ |

^a^ Used to define p24+ gate; in people living with HIV this control is also critical to examine endogenous viral replication. In untreated HIV infection, replicates should be considered.

^b^ See main manuscript text for further details on calculation of replicate number

1. Spin cells down at 500g at room temperature for 5 minutes.
2. Wash cells with 2ml PBS. Spin cells down at 500g at room temperature for 5 minutes and resuspend cells in 100μl PBS.
3. Add 0.2μl Zombie NIR^TM^. Wrap the tube with foil and incubate for 20-30 minutes at room temperature.
4. During this incubation
   1. bring lysolecithin fixative to room temperature
   2. Prepare an ice bucket.
   3. Bring a centrifuge down to 4°C.
5. Add 2ml PBS/tube and centrifuge for 5 minutes at room temperature at 500g. Decant excess supernatant onto paper towel.
6. Fix cells by adding 1ml of 20μg/ml lysolecithin in 4% paraformaldehyde.
7. Vortex. Incubate for 2 minutes at room temperature.
8. Centrifuge for 5 minutes at 4°C at 500g. Decant or aspirate supernatant. Vortex.
9. Add 1ml of cold 50% methanol (-10 to -20°C).
10. Vortex. Incubate on ice for 15 minutes.
11. Centrifuge for 5 minutes at 4°C at 500g. Decant or aspirate supernatant. Vortex.
12. Add 1ml of 0.1% NP-40 (2-8°C).
13. Vortex vigorously. Incubate on ice for 5 minutes.
14. Centrifuge for 5 minutes at 4°C at 500g. Decant or aspirate supernatant. Vortex.
15. Make an antibody mastermix. Per tube add 2ul each of p24, CD3, CD4 and CD8 antibodies.
16. Add antibody mastermix to tubes and vortex. Incubate for 15 minutes at room temperature.
17. Add 2ml of PBS. Centrifuge for 5 minutes at room temperature at 500g. Decant or aspirate supernatant. Vortex.
18. Prepare compensation controls for each fluorochrome.
19. Acquire on a flow cytometer within 6hr.

Gating Scheme:


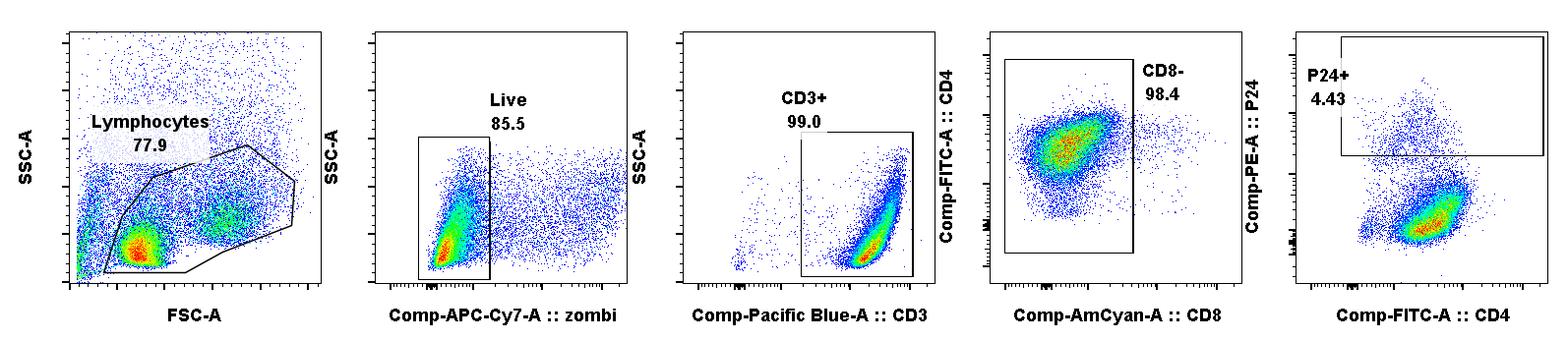


*Note I:* *Acquisition rate <1,000 events/sec is recommended given the low frequency events.*

*Note II: Set CD4+ > 20,000 events or p24% > 500 events in CD4+ target alone as criteria (see main manuscript).*

*Note III: The frequency of infected CD4+ T cells was defined as the percentage of HIV-1 p24 + cells among live, single CD3+ CD8 negative lymphocytes using the uninfected CD4+ cell culture that had a p24+ frequency of < 0.1 across all assays.*
