# Supplementary material for: Reliable Estimation of CD8 T Cell Inhibition of In Vitro HIV-1 Replication
Source: Front Immunol. 2021 Jun 30;12:666991. doi: 10.3389/fimmu.2021.666991 (PMC8278574; doi:10.3389/fimmu.2021.666991)
Supplement: Supplementary file 3 [file DataSheet_2.docx]

**Supplementary Material 2**

**Supplementary Equations**

Recall from the Main Text that the composite frequencies of p24+ cells, in superinfected CD4 T cells co-cultured with CD8 T cells and in CD4 T cells cultured alone, were computed by taking a weighted average of p24+ frequencies in CD4 T cells across all replicates using

$f=\frac{\sum_{i=1}^{n} w_{i}p_{i}}{\sum_{i=1}^{n} w_{i}}=\frac{w_{1}p_{1}+w_{2}p_{2}+\ldots+w_{n}p_{n}}{w_{1}+w_{2}+\ldots+w_{n}},$ (S1)

where the weights, $w_{i}$, are the CD4 T cells in replicates 1 through n, and the frequencies (i.e., proportions or percentages divided by 100), $p_{i}$, are the frequency of p24+ cells in superinfected CD4 T cells in each of these replicates. Equation (S1) is equivalent to taking the sum of all p24+ cells across all replicates and then dividing by the sum of all CD4 T cells across replicates. To produce two separate composite frequencies, this would be done separately for superinfected CD4 T cells cultured alone and for superinfected CD4 T cells co-cultured with CD8 T cells using

$f=\frac{\sum_{i=1}^{n} q_{i}}{\sum_{i=1}^{n} w_{i}}=\frac{q_{1}+q_{2}+\ldots+q_{n}}{w_{1}+w_{2}+\ldots+w_{n}},$ (S2)

where $w_{i}$ are the CD4 T cells in replicates 1 through n, and $q_{i}$ are the number of p24+ cells in superinfected CD4 T cells in each of these replicates.

We present this alternative Equation S2 because it is the equation used in the Excel template “VIA Replicate Calculator” (<https://github.com/glab-hiv/via>). Using either Equation S1 or S2 (without rounding intermediate values) will produce the same composite frequency.
